# Health Care Burden and Expenditure Associated with Adverse Childhood Experiences in Tennessee and Virginia

**DOI:** 10.1007/s40653-021-00390-w

**Published:** 2021-08-18

**Authors:** Glory Okwori, Steven Stewart, Megan Quinn, Delaney Lawson

**Affiliations:** 1 Department of Health Services Management and Policy, East Tennessee State University, Johnson City, TN USA; 2 Department of Biostatistics and Epidemiology, East Tennessee State University, Johnson City, TN USA

**Keywords:** Adverse Child Experiences, Cost, DALYs

## Abstract

To estimate attributable burden and costs of conditions associated with exposure to Adverse Childhood Experiences (ACEs) in Tennessee (TN) and Virginia (VA) during 2017. This is a cross-sectional study of individuals aged 18+ having exposure to ACEs using Behavioral Risk Factor Surveillance System (BRFSS) data. Eight chronic diseases (asthma, obesity, hypertension, diabetes, chronic obstructive pulmonary disease (COPD), depression, cardiovascular disease, and arthritis) and two risk factors (smoking and drinking) associated with ACEs were analyzed. Pearson's chi-square tests analyzed the association between ACEs, risk factors and chronic diseases. The population attributable risks (PAR) were estimated for the ACEs related diseases and risk factors and combined with health care expenses and Disability Adjusted-Life-Years (DALYs). Among those who experienced at least 1 ACE in TN, 10% had COPD, 17% had diabetes, 36% had obesity, and 30% had depression. Individuals who experienced at least 1 ACE in VA had higher percentages for COPD, obesity and depression diseases compared to those who had no ACE (*p*< .0001). ACEs’ exposure resulted in a burden of about 115,000 years and 127,000 years in terms of DALYs in TN and VA, respectively. The total health spending associated with ACEs based on PARs was about $647 million ($165 per adult) and $942 million ($292 per adult) in TN and VA respectively. The total costs associated with ACEs was about $15.5 billion ($3948) per person) and $20.2 billion ($6288 per person) in TN and VA, respectively. This study emphasizes the need to reduce ACEs due to high health and financial costs.

## Introduction

Adverse childhood experiences (ACEs) have been considered a significant risk factor for poorer adult outcomes since the original ACEs study conducted by Felitti et al. asserted a gradient correlation between exposure to ACEs and negative adulthood health outcomes including various chronic conditions. ACEs include exposure to physical, emotional, or sexual abuse and household dysfunction prior to one’s 18th birthday (Felitti et al., 1998). ACEs have been shown to be associated with problems such as heart disease, poor mental health, poor emotional health, suicide, depression, physical inactivity, obesity, smoking, substance abuse and other chronic diseases (Chang et al., 2019; Felitti et al., 1998). Researchers continue to study the pervasive and persistent effects of ACEs in a variety of settings that has further validated the link between ACEs and less favorable adulthood outcomes (Clemens et al., 2018; McCrory et al., 2015).

Increasing prevalence and rising cost of treatment for chronic disease conditions led to research studies linking ACEs to chronic diseases in adulthood, which are suspected to contribute to the significant economic burden in the United States (Felitti et al., 1998; Peterson et al., 2018; Raghupathi & Raghupathi, 2018). The economic impact for many conditions is not limited to medical expenses; the burden also includes lost wages and revenue caused by absenteeism, premature mortality, and diminished educational attainment (Fang et al., 2011; Raghupathi & Raghupathi, 2018). Furthermore, childhood traumas can diminish average educational attainment economic accomplishments such as employment status, occupation level, and annual income (Currie & Widom, 2010).

Previous research has varied widely on the projected total economic burden and medical costs of individual and aggregate ACEs. Holmes et al. estimated the lifetime medical cost (defined here as the costs between ages 20 and 64) of childhood exposure to domestic violence in 2016 US dollars as over $12 billion nationally (Holmes et al., 2018). Similarly, the estimated adult lifetime medical costs (ages 1-64) of sexual abuse alone was estimated at $399 million in 2015 U.S. dollars (Letourneau et al., 2018). The 2012 study by Fang et al. showed that aggregate childhood maltreatment (defined here as neglect, physical abuse, emotional abuse, and sexual abuse) resulted in a total economic burden of over $120 billion dollars in 2010 U.S. dollars, over $18 billion in short-term health care costs, $32,648 per person, and over $6 billion in long-term health care costs, $10,530 per person (Fang et al., 2011). A follow up to the Fang et al. study that used a more robust model to calculate the total economic burden and adjusted for costs in 2015 presented national lifetime cost of childhood maltreatment of $48 billion; $35,162 per person in short term health care costs and $11,341 in long-term health care costs (Peterson et al., 2018).

This study provides further information to previous research, such as the study conducted by the Sycamore Institute which found that the direct costs of medical care and lost productivity due to ACEs was $5.2 billion in Tennessee (Sycamore Institute, n.d.). This study is original in that researchers describe a regional burden of ACEs rather than the cost of a single state by analyzing data from both Tennessee (TN) and Virginia (VA). These states were selected because of their proximity and affiliation to authors in this study and the high prevalence of ACEs (Bethell et al., 2017). These states also had all the necessary publicly available data required for the analyses. A similar analysis was completed in California (CA) utilizing BRFSS data from 2008, 2009, 2011, and 2013 as well as 2016 global burden of disease (GBD) and DALYs. The economic burden per exposed adult was $5,769 in 2017 dollars (Miller et al., 2020). In 2016, the national average of children younger than 18 years of age that experienced at least one ACE was 46.3%; the reported prevalence in TN was 48.1% and in VA was 41.2%.Click or tap here to enter text. Outcome variables and risk factors for this study were selected because of their correlation to ACEs and use in previous studies (Downey et al., 2017; Miller et al., 2020). The aim of this study was to estimate the health care costs and disease burden associated with exposure to ACEs in TN and VA.

## Methods

### Study Population & Study Design

Cross-sectional data were obtained from the 2017 Tennessee (TN) and Virginia (VA) Behavioral Risk Factor Surveillance System (BRFSS). BFRSS is a random digit telephone survey delivered by health departments and the Centers for Disease Control and Prevention (CDC) collaboratively primarily focusing on chronic diseases among adults. Eligibility was limited to non-institutionalized adults aged 18 years or older. The sample was weighted to ensure that the data are representative of the population in TN and VA and to adjust for demographic characteristics (Centers for Disease Control and Prevention, 2019). The states’ health departments surveyed residents regarding demographics, chronic diseases and risk behaviors utilizing a standard questionnaire and some states include additional modules such as ACEs as well as any other state-developed questions of interest (TN Department of Health, 2020; Virginia Department of Health, 2020). This study used the 2017 BRFSS data from the TN and VA Departments of Health. This study was exempt from the East Tennessee State University Institutional Review Board.

### Measures

Binary responses to self-reported chronic disease variables (yes/no), were used in this analysis. Adults that responded yes to ever having the following eight chronic conditions were examined in this study: asthma, obesity [high body mass index (BMI)], hypertension, diabetes, chronic obstructive pulmonary disease (COPD), depression, cardiovascular disease, and arthritis. Disability-adjusted life years estimates for arthritis were measured as rheumatoid arthritis, rheumatoid arthritis and gout.

ACEs were examined as binary responses (yes/no or having experienced at least once/never) to the CDC’s ACEs module. The decision to use 0 ACE vs. 1+ ACE was based on lack of definitive information from prior studies to compare how ACEs scores were dichotomized. Researchers have mentioned that four or more ACEs have a higher probability of adverse outcomes (Felitti et al., 1998; McKelvey et al., 2016). However, these studies used the full set of ACEs questions. Thus, the data was examined as continuous and then the cutoff threshold was determined based on the data. From a different article that examined health burden in California using BFRSS data, most of the health burden resulted from exposure to fewer ACEs and they questioned the strategy of focusing on 4 or more ACEs (Miller et al., 2020).

ACEs from this module totaled 11 questions regarding participants’ childhood before 18 years (Cronholm et al., 2015). The questions included physical or emotional abuse, sexual abuse, household substance abuse, household mental illness, incarceration of family members, parental divorce or domestic violence (Centers for Disease Control and Prevention, 2019). Participants received 1 point per question if they responded yes or at least once to each question and the aggregate ACEs scores were calculated across the 11 questions. The ACEs scale utilized in this study has been shown to have adequate test reliability and good retrospective reporting (Dube et al., 2004).

The risk factors included smoking (at least 100 cigarettes in a lifetime) and the use of alcohol (at least one drink in the past 30 days). Prior studies that used the BFRSS data and examined the association between ACEs and health outcomes utilized this measure of smoking and found that smoking was associated with heart disease and poor health (Dong et al., 2004; Dube et al., 2010). A study that examined the use of alcohol defined current drinking as having at least one drink in the past 30 days which correlated with poor health and participants that reported current drinking also reported the use of substances such as tobacco and marijuana (England et al., 2020). Alcohol use has been shown to be a leading risk factor for the global burden of disease and a study examined the overall health effect due to alcohol consumption by generating improved estimates of alcohol attributable deaths and DALYs (Griswold et al., 2018). The results showed that the total attributable burden of alcohol use was greater than past evidence and the amount of consumption that reduces health loss is zero (Griswold et al., 2018).

### Statistical Analyses

The sample was weighted to ensure that the data is representative of the population in TN and VA and to adjust for demographic characteristics. Descriptive statistics were first conducted for ACEs and chronic diseases individually. Cross tabulation estimates were then calculated to determine the association between ACEs, chronic diseases and risk behaviors for both TN and VA using Pearson's chi-square tests. Data analyses were completed in SAS 9.4 in July of 2020 (Version 9.4. SAS Institute Inc., Cary, NC, USA).

Two-way tables were created comparing ACEs exposure with the chronic diseases and risk factors. The relative risks with 95% confidence intervals were calculated by using the following formula $RR\left(95\%CI\right)=\frac{\frac{exposed\;with\;outcome}{total\;exposed}}{\frac{not\;exposed\;with\;outcome}{total\;not\;exposed}}$. The attributable risk (AR), used to describe the sample, was calculated using the following: $AR=\frac{exposed\;with\;outcome}{total\;exposed}-\frac{not\;exposed\;with\;outcome}{total\;not\;exposed}$. This was then used to determine the attributable risk percent using the formula $AR\%=\frac{AR}{(\frac{exposed\;with\;outcome}{total\;exposed})}$. The attributable risk percents were interpreted to be the percent of the outcome in the sample group that could have been prevented if the exposures were eliminated. The population attributable risk (PAR), used to generalize findings to the entire population, was calculated using the following formula $PAR=\frac{total\;with\;outcome}{population}-\frac{(\frac{not\;exposed}{total\;with\;outcome})}{total\;unexposed}$ (University of California San Francisco, 2013). The PAR was then divided by the proportion of the disease in the population to determine the population attributable risk percent. The population attributable risk percent was interpreted as the percent of cases of the outcome in the population attributable to the exposure.

Disability Adjusted Life Years (DALYs) are defined as the sum of years of healthy lives lost (YLL) and years of lives lost due to mortality (YLD). Recommendations for DALYs calculation were followed which include the use of a prevalence-based approach, standard life tables, adjustment for comorbidity and databases with default datasets to improve consistencies in DALY-based burden of diseases studies (Devleesschauwer et al., 2014; Murray et al., 2012). Frequencies were obtained (weighted) from the 2017 Global Burden of Disease (GBD) study for TN and VA (Institute for Health Metrics and Evaluation, 2020). The prevalence-based DALY estimates for individuals aged 20 years and older (the only data for adults) were multiplied by the excess risk due to ACEs to obtain estimates of the health burden associated with ACEs.

Published estimates of nationally representative data on various ACE related conditions in 2016 were utilized to estimate spending for participants in this study and inflated to 2017 dollars (Bureau of Economic Analysis (BEA), 2020; Waehrer et al., 2020). The statewide share of the US personal healthcare expenses in the national health expenditure data were used to calculate adult health care costs. The costs were obtained by multiplying estimates by 0.019 and 0.025, the share of the US personal health expenses for 2017 for TN and VA, respectively (Lassman et al., 2017). The average annual growth rate was used to provide estimates for 2017. The aggregated costs of the chronic diseases were divided by the number of persons aged 18 and older exposed to ACEs (78% of the 6.7 million people in TN or 78% of the 8.5 million people in Virginia times the percent of individuals exposed to ACEs according to BFRSS).

## Results

The study sample consisted of 5,843 respondents in TN and 9,630 respondents in VA. In TN, among those who had experienced at least 1 ACE, 10% had COPD, 17% had diabetes, 36% had obesity, and 30% had depression, while among those who had no ACE, 9% had COPD, 13% had diabetes, 32% had obesity, and 15% had depression (*p*< .0001) (Table 1). While in VA, individuals who had experienced at least 1 ACE had significantly higher percentages than those who had no ACE for COPD, obesity and depression diseases (*p*< .0001).

**Table 1:** Prevalence of aces count and risk behaviors by chronic diseases among adults living in TN and VA, 2017

	**Arthritis**		**Asthma**		**COPD**		**Diabetes**		**Obesity**		**CVD**		**Depression**		**Hypertension**	
	**A**	**B**	**A**	**B**	**A**	**B**	**A**	**B**	**A**	**B**	**A**	**B**	**A**	**B**	**A**	**B**
**Tennessee (N=5,843)**																
***ACEs***		****		****		****		****		****		****		****		****
0	723 (38.62)	33.23	157 (69.47)	70.13	188 (10.06)	8.99	327 (17.87)	13.13	551 (31.38)	31.79	208 (11.19)	10.13	762 (33.26)	14.55	932 (49.84)	43.68
≥1	912 (39.79)	32.47	274 (65.87)	55.94	331 (14.37)	10.42	386 (17.24)	16.52	810 (37.26)	35.75	256 (11.21)	8.67	293 (15.64)	30.33	1067 (46.33)	39.77
***Risk behaviors***																
**Ever smoked 100 cigarettes**		****		****		****		****		****		****		****		****
Yes	1159 (43.79)	37.73	322 (71.56)	64.75	546 (20.60)	16.37	476 (18.40)	16.61	800 (31.60)	31.05	368 (14.00)	12.64	826 (31.16)	29.85	1319 (49.77)	43.6
No	896 (30.96)	24.29	246 (62.76)	57.4	131 (4.52)	3.68	404 (14.27)	10.44	960 (35.77)	34.38	211 (7.31)	5.14	528 (18.28)	17.01	1207 (41.58)	34.76
**Drank alcohol in past 30 days**		****		****		****		****		****		****		****		****
Yes	644 (28.38)	22.55	181 (59.93)	52.31	167 (7.33)	5.43	219 (9.82)	7.58	675 (30.86)	30.59	139 (6.11)	4.82	482 (21.23)	19.91	824 (36.19)	30.16
No	1375 (43.60)	37.56	372 (71.40)	68.05	497 (15.73)	13	646 (21.02)	18.28	1046 (35.82)	34.85	429 (13.74)	11.75	855 (27.12)	25.92	1646 (52.06)	46.41
**Virginia (N=9,630)**																
***ACEs***				****		****		****		****		****		****		****
0	1179 (34.49)	26.33	223 (69.69)	68.67	229 (6.68)	4.98	528 (15.72)	12.32	885 (27.85)	25.41	308 (9.02)	7.08	407 (11.88)	11.45	1474 (42.97)	34.32
≥1	1661 (35.81)	26.38	490 (65.77)	61.11	471 (10.15)	8.38	676 (14.98)	10.89	1528 (34.84)	34.59	398 (8.61)	6.74	1219 (26.28)	25.09	1920 (41.27)	32.91
***Risk behaviors***																
**Ever smoked 100 cigarettes**		****		****		****		****		****		****		****		****
Yes	1571 (39.62)	31.53	408 (72.21)	70.55	592 (14.91)	12.88	641 (16.57)	13.49	1203 (31.88)	33.13	488 (12.35)	10.76	973 (24.51)	24.94	1834 (46.02)	39.90
No	1542 (29.23)	20.74	434 (64.30)	60.25	197 (3.73)	2.88	666 (12.89)	8.9	1488 (30.60)	27.95	293 (5.56)	3.84	867 (16.42)	15.44	1920 (36.35)	27.13
**Drank alcohol in past 30 days**		****		****		****		****		****		****		****		****
Yes	1378 (28.11)	21.32	369 (61.09)	58.47	325 (6.62)	4.98	432 (9.01)	6.69	1294 (27.57)	27.84	302 (6.17)	4.60	902 (18.39)	18.30	1697 (34.56)	28.23
No	1708 (40.52)	30.19	463 (73.96)	70.76	457 (10.82)	9.47	867 (21.07)	16.01	1371 (35.68)	33.06	471 (11.21)	9.13	919 (21.76)	20.86	2014 (47.58)	37.21

*P*-value obtained from Pearson Chi-square analysis, *A* Unweighted frequency and percent, *B* Weighted percent

^*^P<0.05; **P<0.01; ***P<0.001; ****P<0.0001

In Tennessee, among individuals who had smoked at least 100 cigarettes in their entire lives, 38% had arthritis, 65% had asthma, 16% had COPD, 17% had diabetes, 13% had CVD, 30% had depression, and 44% had hypertension (*p*< .0001) (Table 1). While in VA, individuals who had smoked at least 100 cigarettes in their entire lives had significantly higher percentages than those who had not smoked for all eight chronic diseases (*p*< .0001). However, in TN and VA, the prevalence of all eight chronic diseases was lower among individuals who drank alcohol within the past 30 days compared to those who did not (*p*< .0001).

In Tennessee, among individuals who had experienced forced sex, 53% had arthritis, 65% had asthma, 23% had COPD, 19% had diabetes, 49% had obesity, 11% had CVD, 58% had depression and 49% had depression (*p*< .0001) (Table 2). In TN, among individuals who had experienced physical abuse, 38% had arthritis, 64% had asthma, 16% had COPD, 16% had diabetes, 38% had obesity, 11% had CVD, 48% had depression and 42% had depression (*p*< .0001). In TN, among children who lived with alcoholic parents, 39% had arthritis, 54% had asthma, 15% had COPD, 15% had diabetes, 31% had obesity, 10% had CVD, 38% had depression and 45% had depression (*p*< .0001). In Virginia, among individuals who had experienced forced sex, 49% had arthritis, 76% had asthma, 19% had COPD, 20% had diabetes, 48% had obesity, 11% had CVD, 52% had depression and 44% had depression (*p*< .0001) (Table 2). In VA, among individuals who had experienced physical abuse, 42% had arthritis, 64% had asthma, 13% had COPD, 16% had diabetes, 39% had obesity, 9% had CVD, 36% had depression and 42% had depression (*p*< .0001). In VA, among children who lived with alcoholic parents, 41% had arthritis, 67% had asthma, 14% had COPD, 17% had diabetes, 36% had obesity, 10% had CVD, 32% had depression and 44% had depression (*p*< .0001).

**Table 2:** Prevalence of ace types by chronic diseases among adults living in TN and VA, 2017

	**Arthritis**	**Asthma**	**COPD**	**Diabetes**	**Obesity**	**CVD**	**Depression**	**Hypertension**
	N^a^ (%)^b^	N (%)	N (%)	N (%)	N (%)	N (%)	N (%)	N (%)
**Tennessee (N=5,843)**								
***ACE Types***								
	****	****	****	****	****	****	****	****
Sworn at/insulted	452 (33.95)	161 (58.14)	177 (11.62)	178 (13.29)	406 (35.39)	117 (7.87)	458 (39.05)	495 (38.49)
	****	****	****	****	****	****	****	****
Physically hurt	278 (37.7)	97 (64.28)	125 (16.2)	118 (15.9)	235 (38.4)	80 (10.56)	290 (47.95)	285 (41.72)
	****	****	****	****	****	****	****	****
Forced to touch sexually	167 (46.82)	55 (62.61)	67 (15.69)	61 (18.48)	138 (48.01)	39 (9.83)	174 (52.58)	168 (45.34)
	****	****	****	****	****	****	****	****
Forced touch sexually	211 (47.35)	68 (63.57)	86 (17.32)	73 (17.95)	166 (44.57)	46 (8.43)	222 (53.03)	216 (46.08)
	****	****	****	****	****	****	****	****
Forced sex	122 (52.72)	46 (64.7)	60 (22.82)	44 (18.95)	88 (48.75)	31 (11.16)	126 (57.93)	116 (48.79)
	****	****	****	****	****	****	****	****
Witnessed parents beat each other up	307 (36.2)	107 (59.93)	135 (14.48)	131 (13.4)	242 (32.72)	89 (10.31)	290 (40.44)	337 (41.78)
	****	****	****	****	****	****	****	****
Lived with parent/guardian who was depressed	266 (33.06)	106 (57.76)	102 (11.65)	100 (12.08)	236 (35.62)	57 (7.48)	346 (50.1)	290 (38.04)
	****	****	****	****	****	****	****	****
Lived with alcoholic	427 (39.36)	123 (53.95)	188 (15.32)	170 (14.83)	314 (31.8)	117 (10.32)	373 (37.6)	470 (45.04)
	****	****	****	****	****	****	****	****
Parent/guardian who abused drugs	142 (30.93)	53 (53.3)	59 (10.59)	48 (9.26)	128 (36.22)	35 (7.85)	170 (43.04)	145 (36.69)
	****	****	****	****	****	****	****	****
Parent/guardian imprisoned	116 (29.92)	43 (47.1)	51 (9.07)	50 (12.28)	99 (33.29)	29 (5.55)	129 (40.8)	134 (40.56)
	****	****	****	****	****	****	****	****
Parents divorced	390 (25.4)	136 (49.52)	152 (8.89)	178 (12.04)	411 (38.52)	114 (7.08)	353 (28.07)	474 (34.58)
**Virginia (N=9,630)**								
	****	****	****	****	****	****	****	****
Sworn at/insulted	834 (34.81)	255 (63.91)	249 (10.38)	321 (13.74)	773 (33.90)	186 (7.78)	746 (31.10)	917 (38.10)
	****	****	****	****	****	****	****	****
Physically hurt	468 (42.01)	133 (64.25)	147 (13.18)	175 (16.25)	406 (38.93)	104 (9.35)	406 (36.31)	473 (42.31)
	****	****	****	****	****	****	****	****
Forced to touch sexually	252 (44.13)	98 (70.00)	84 (14.71)	96 (17.65)	232 (42.57)	48 (8.42)	264 (46.07)	234 (40.91)
	****	****	****	****	****	****	****	****
Forced touch sexually	355 (44.99)	122 (67.78)	129 (16.43)	135 (18.02)	299 (40.24)	70 (8.93)	363 (46.12)	336 (42.64)
	****	****	****	****	****	****	****	****
Forced sex	171 (49.14)	62 (75.61)	67 (19.25)	66 (20.18)	155 (47.69)	40 (11.63)	181 (52.16)	152 (43.68)
	****	****	****	****	****	****	****	****
Witnessed parents beat each other up	458 (38.65)	131 (63.90)	149 (12.58)	197 (17.10)	423 (37.80)	109 (9.19)	378 (31.76)	505 (42.47)
	****	****	****	****	****	****	****	****
Lived with parent/guardian who was depressed	456 (38.71)	158 (63.97)	125 (10.64)	141 (12.41)	370 (33.21)	91 (7.77)	555 (47.15)	418 (35.42)
	****	****	****	****	****	****	****	****
Lived with alcoholic	715 (40.97)	196 (67.12)	237 (13.57)	284 (16.77)	595 (35.91)	180 (10.35)	566 (32.47)	777 (44.37)
	****	****	****	****	****	****	****	****
Parent/guardian who abused drugs	225 (35.05)	78 (60.00)	72 (11.18)	83 (13.37)	231 (37.32)	49 (7.62)	224 (34.89)	228 (35.35)
	****	****	****	****	****	****	****	****
Parent/guardian imprisoned	166 (35.78)	56 (63.64)	59 (12.66)	60 (13.19)	162 (36.08)	44 (9.54)	137 (29.65)	169 (36.34)
	****	****	****	****	****	****	****	****
Parents divorced	622 (32.38)	217 (62.00)	173 (9.01)	259 (13.89)	672 (36.80)	135 (7.06)	489 (25.43)	741 (38.55)

*P*-value obtained from Pearson Chi-square analysis

^*^P<0.05; **P<0.01; ***P<0.001;****P<0.0001

^a^Unweighted frequencies

^b^Weighted percent

Table 3 presents PAR results. Any AR and PAR at or below 0 was interpreted to be no risk. In TN and VA, there was no risk due to ACEs for asthma, diabetes, cardiovascular disease, or hypertension. However, ACEs were associated with an increased risk of smoking and alcohol use in both TN and VA. In TN, 11% of cases of smoking and 11% of cases of alcohol use were associated with ACEs. In VA, 12% of cases of smoking and 7% of cases of alcohol use were associated with ACEs.

**Table 3:** Attributable risk (AR) & population attributable risk (PAR)

	**Arthritis**	**Asthma**	**COPD**	**Diabetes**	**Obesity**	**CVD**	**Depression**	**Hypertension**	**Smoking**	**Drinking**
	**AR** **(PAR)** **(%)**	**AR** **(PAR)** **(%)**	**AR** **(PAR)** **(%)**	**AR** **(PAR)** **(%)**	**AR** **(PAR)** **(%)**	**AR** **(PAR) (%)**	**AR** **(PAR)** **(%)**	**AR** **(PAR)** **(%)**	**AR** **(PAR)** **(%)**	**AR** **(PAR)** **(%)**
**Tennessee**										
***ACEs***	2.94 (1.64)	-5.47 (-3.48)	29.98 (19.11)	-3.65 (-1.98)	15.78 (9.39)	0.16 (0.09)	52.97 (38.26)	-7.57 (-4.04)	18.98 (11.44)	18.00 (10.82)
**Virginia**										
***ACEs***	3.66 (2.14)	-5.95 (-4.09)	34.21 (23.02)	-4.96 (-2.79)	20.07 (12.71)	-4.77 (-2.69)	54.80 (41.09)	-4.12 (-2.33)	18.98 (11.89)	11.29 (6.82)

In TN, increased risk of arthritis, COPD, obesity, and depression was associated with having experienced one or more ACEs (Table 3). The greatest association between ACEs and the health outcomes was with depression, which occurred at twice the risk. In TN, 38% of cases of depression are associated with ACEs, and 53% of cases of depression in our sample could have been prevented if ACEs were eliminated. In VA, ACEs were associated with an increased risk of having arthritis, COPD, obesity, and depression. In VA, 2% of cases of arthritis, 23% of cases of COPD, 13% of cases of obesity and 41% of cases of depression may be attributed to ACEs. The greatest increase in risk associated with ACEs was for depression; 54% of cases of depression could have been prevented by eliminating ACEs.

Table 4 presents total DALYs (in years) for each outcome or risk factor for each state, health burden (in years) which is the portion of the DALYs attributable to ACEs exposure, total cost for an outcome or risk factor for the state, and costs or spending associated with exposure to ACEs. Of the conditions considered in this model; arthritis, COPD, depression, obesity, smoking, and alcohol use yielded positive population attributable risks (PARs). Other conditions had negative PARs which were considered as zero to avoid overestimating. Multiplying the PARs by the DALYs yielded the burden attributable to ACEs and multiplying the PAR by the total costs yielded the costs that results from ACEs exposure.

**Table 4:** Health burden and spending associated with aces

**ACEs Condition**	**DALYs (years)**		Health Burden (PAR* DALYs)		**Cost($)**		Health Spending (PAR* Cost)	
	TN	VA	TN	VA	TN	VA	TN	VA
**Chronic Diseases**								
**Asthma**								
ACEs Exposure (≥1)	9,463	13,449	-	-	533,157,233	701,522,676	-	-
**Arthritis**^**a**^								
Rheumatoid Arthritis	6,278	6,903			535,646,181	704,797,607		
Osteoarthritis	21,498	25,602			1,542,337,727	2,029,391,746		
Gout	3,095	4,178			70,957,682	53,927,839		
ACEs Exposure (≥1)	30,872	36,684	506	785	2,148,941,590	2,788,117,191	35,242,642	59,665,708
**COPD**								
ACEs Exposure (≥1)	126,370	105,275	24,149	24,234	657,309,637	864,881,101	125,611,872	199,095,629
**CVD**								
ACEs Exposure (≥1)	410,557	372,573	-	-	4,834,521,227	6,361,212,141	-	-
**Depression**								
ACEs Exposure (≥1)	50,158	58,961	19,190	24,227	1,171,212,711	1,541,069,357	448,105,983	633,225,399
**Diabetes**								
ACEs Exposure (≥1)	89,739	99,575	-	-	2,090,253,205	2,750,333,164	-	-
**Hypertension**								
ACEs Exposure (≥1)	24,046	14,144	-	-	1,512,397,096	1,989,996,179	-	-
**Obesity**								
ACEs Exposure (≥1)	291,234	288,763	27,347	36,702	183,455,289	241,388,538	17,226,452	30,680,483
**Risk Factors**								
**Smoking**								
ACEs Exposure (≥1)	290,635	288,763	33,249	34,334	35,824,650	47,137,697	4,098,340	5,604,672
**Alcohol use**								
ACEs Exposure (≥1)	95,127	106,032	10,293	7,231	153,979,287	202,604,325	16,660,559	13,817,615
**ALL OUTCOMES (Total)**	1,449,072	1,420,902	114,734	127,513	15,469,993,515	20,276,379,560	646,945,848	942,089,507

^*^Negative PARs had 0 allocated to them

^**a**^DALYs and cost of arthritis was the calculated as the sum of the 3 types of arthritis

In terms of DALYs, exposure to ACEs resulted in a burden of nearly 115,000 years in TN and over 125,000 years in VA. Smoking represented the combined highest burden in years (TN – 33,248, VA – 34,333), followed by obesity (TN – 27,346, VA – 36,701), COPD (TN – 24,149, VA – 24,234), depression (TN – 19,190, VA – 24,227), alcohol use (TN – 10,292, VA – 7,231), and arthritis (TN – 506, VA – 785). The health spending of all outcomes attributable to ACEs cost TN nearly 650 million dollars and VA nearly $942 million. Depression accounted for the largest combined sum of health care spending (TN - $448,105,983, VA – $633,225,398) followed by COPD (TN – $125,611,871, VA - $199,095,629), arthritis (TN - $35,242,642, VA - $59,665,707), obesity (TN – $17,226,451, VA – 30,680,483), alcohol use (TN - $16,660,558, VA – $13,817,614), and smoking (TN - $4,098,339, VA – $5,604,672).

Table 5 shows the per capita costs of ACEs for each state. To obtain the exposed population, the total population of each state was multiplied by the percent of the population exposed to one or more ACEs. The ACEs attributable burden in years (DALYs) and dollars were divided by the population exposed. TN had an adult population of 6,630,000; the prevalence of exposure to ACEs was 59.1%, thus the adult population exposed was 3,918,330. With DALYs valued at $235,855 in 2017 dollars, the monetized cost of DALYs attributable to ACEs was over $27 billion, $6906 per exposed adult Tennessean. The total health spending attributable to ACEs (derived from PARs) was nearly $650 million in TN, $165 per exposed adult Tennessean. The total cost of ACEs related to these outcomes in TN was nearly $15.5 billion, which can be estimated as $3948 per exposed adult Tennessean.

**Table 5:** Cost per person of aces conditions

**ALL OUTCOMES**	**Exposure**	**Adults exposed to ACES**	**Total Burden (years)**	**Total Burden ($)**^**a**^	**Total Burden** **/person($)**	**Total Health Spending ($)**	**Health Spending per person ($)**	**Total** **Cost**	**Health** **Cost per person ($)**
**Tennessee**									
Adult Population ( >18)	6,630,000								
ACEs Exposure (≥1)	59.1%	3,918,330	114,734	27,060,657,657	6,906	646,945,847	165	15,469,993,515	3,948
**Virginia**									
Adult Population ( >18)	5,226,000								
ACEs Exposure (≥1)	61.7%	3,224,442	127,513	30,074,684,112	9,327	942,089,506	292	20,276,379,559	6,288

^**a**^Value of a DALY $235,855 (2017)

Virginia had an adult population of 5,226,000 with a prevalence of exposure to ACEs of 61.7% resulting in an exposed adult population of 3,224,442. The monetized loss due to DALYs represents over $30 billion (in 2017 dollars), $9,327 per exposed adult in VA. Similarly, the total health spending attributable to ACEs (derived from PARs) was over $942 million, $292 per exposed adult. The total cost of ACEs for these outcomes exceeded $20 billion in VA which can be estimated as $6,288 per exposed adult Virginian.

## Discussion

This study provides the first examination of the health burden and costs associated with ACEs in two states, TN and VA, providing a regional perspective of the health burden and costs associated with ACEs, which is a novel contribution. ACEs have been shown to have multifaceted effects on future health ranging from increased incidence of cancer to the perpetuation of violence and increased risk of teenage pregnancy (Hughes et al., 2017). Using 2017 BRFSS data from TN and VA, this study aimed to show the economic impact of exposure to any ACE to an associated medical cost or DALYs burden for several adulthood health outcomes, health risk factors, and chronic health conditions. Consistent with other studies, the current study found attributable risks due to exposure to any ACEs for arthritis, COPD, depression, obesity, smoking, and alcohol use (Miller et al., 2020; Sidmore, 2018; Sycamore Institute, n.d.). An analysis of 2013 BRFSS data by Sidmore showed a population attributable risk (PAR) between ACEs and frequent mental distress, COPD, emphysema, chronic bronchitis, increased days of poor physical health, smoking, asthma (current or former), diabetes, heavy alcohol consumption, obesity, increased activity limitation, diagnosis of arthritis, insufficient sleep, and separation or divorce in Alaska (Sidmore, 2018). Previous research has linked exposure to ACEs with increased risks for asthma, cardiovascular diseases, diabetes, and hypertension, however, when looking at the attributable risk for these outcomes in the study population, there was no statistically significant relationship.

Sexual abuse was reported more often among individuals with asthma, obesity, depression and hypertension. Research has shown that persons with a history of childhood sexual abuse are more likely to experience poorer cardiopulmonary health compared to persons without childhood sexual abuse (Hulme, 2000). Also, research has demonstrated that childhood sexual abuse is associated with obesity (BMI≥30)(Aaron & Hughes, 2007). A prospective longitudinal study examined the developmental changes in BMI among girls with and without childhood sexual abuse and showed that the two groups did not vary significantly in BMI until early adulthood (Noll et al., 2007). Depression is often reported by adults who have experienced childhood sexual abuse and may impact health symptoms through health behaviors (Irish et al., 2010).

Physical abuse had the highest prevalence among persons with asthma. Consistent with these findings, physical abuse was linked to asthma in a prospective study conducted on African American women and this association was stronger than that of asthma and sexual abuse (Coogan et al., 2013). A case control study of children showed that physical or sexual abuse was linked to asthma, health care use for asthma, and medications for asthma even after accounting for health insurance, income and other confounders.

Medical conditions attributable to childhood traumas can result in increased utilization of hospital services, psychiatric care, as well as care related to chronic conditions, which includes increased primary and specialty care utilization as well as the cost of medications (Fang et al., 2011). While medical expenses represent a significant burden, reduced quality of life resulting from ACE attributable conditions also represents a burden in disability adjusted life years (DALYs) and premature morbidity. Previous research has utilized a variety of methods to calculate lifetime costs of ACEs based upon costs of health care and absenteeism in a single state, healthcare costs and DALYs correlating to several chronic conditions for a single state, or estimated national costs for childhood maltreatment (Miller et al., 2020; Sidmore, 2018).

Lifetime projected costs have varied widely among different models. The costs of childhood maltreatment in 2010 was estimated at $120 billion, and a model proposed that the 2015 costs exceeded $428 billion (Peterson et al., 2018). Similarly, even the costs of a single ACE have been expensive; in 2016, the medical costs resulting from sexual abuse alone cost the U.S. nearly $400 million dollars, medical costs attributed to exposure to domestic violence exceeded $12 billion (Letourneau et al., 2018).

The current study found a total burden of ACEs in TN of over $27 billion, $6,906 per exposed adult. The economic burden in VA exceeded $30 billion, $9,327 per exposed adult. The differences between the current study and the study done in CA could be due to disparities in costs between the states; the CA study did not calculate DALYs for drinking, smoking or obesity; and the current study could not calculate attributable risk for asthma or CVD for the population studied due to data limitations. The prevalence of exposure to ACEs was similar in the three states; CA 61%, (Miller et al., 2020), TN 59.1%, and VA 61.7%.

Whichever method of analysis researchers utilize, and even after accounting for the differences between the current and previous studies, the consistent finding is that ACEs represent a significant economic burden, in terms of costs and DALYs. Furthermore, considering ways to prevent or mitigate the effects of ACEs can become complicated, for many forms of childhood trauma, exposure increases the likelihood of perpetuating similar traumas to the next generation (Holmes et al., 2018).

As resources are limited and the burden of ACEs shown here is very high, there is the need for a wide range of prevention and treatment strategies to mitigate these effects. Some studies have examined the efficacy of screening for adversities during childhood in primary care, and have shown beneficial results related to costs, quality, feasibility, and reduced prevalence of ACEs (Eismann et al., 2019; Gillespie & Folger, 2017). For example, the SEEK program (Safe Environment for Every Kid) is a primary care-based intervention which helps to identify and address psychosocial risk factors for child maltreatment as well as prevent maltreatment. Importantly, the model has been shown to be cost saving. The costs of maltreatment averted were significantly less than the short- or long-term costs of mental and medical care as well social services. The model saved approximately $2,151, 878 health care costs for over 29,000 children (Lane et al., 2021).

Screening practices which do not rely solely on specific ACEs but are based on current or historical traumatic events and chronic stress could be helpful. Due to the large number of relevant ACEs that can be added to a screening tool, and the varying impact across developmental age groups, such practices may be relevant when the goals for screening are to identify children experiencing trauma and tailor interventions to achieve good health. ACE screening can elicit different emotional reactions which could cause distress since patients reflect on negative experiences in their lives (American Academy of Pediatrics, n.d.). Thus, health care workers should administer screenings using trauma-informed methods that avoids re-traumatization.

Suggested mechanisms for the perpetuation of abuses include individuals who have been abused being more likely to socially isolate and more likely to respond aggressively to various stressors from their offspring (Holmes et al., 2018). Alternatively, it has been suggested that if a person develops resilience before exposure to a moderate ACE the effect could be attenuated (Crandall et al., 2019). Furthermore, if an individual concurrently experiences mild ACEs alongside experiences that encourage productive adulthood outcomes; the ACEs could contribute to adulthood resilience, mitigating some of the negative effects (Crandall et al., 2019).

Researchers have identified resilient or protective factors to include having a sense of belonging, protective community and personal abilities (Narayan et al., 2018). Other traits of resiliency have been identified as high self-esteem, internal locus of control, cognitive reappraisal, determination and social competence (Bellis et al., 2018). A study showed that the strength of the association between the prevalence of flourishing and resilient measures were comparable among children exposed to various levels of ACEs (Bethell et al., 2019).

Programs that aim to reduce stress through practices such as meditation and increasing self-awareness have been shown to be beneficial. Some of these programs have shown reduction in anxiety, depressive symptoms, hostility, and allostatic load as well as increased attention and sense of well-being and more stable cortisol levels (Ortiz & Sibinga, 2017). These programs reduced the negative impact of ACEs and improved outcomes, thus potentially decreasing poor health outcomes during adulthood.

Community based programs benefit from disseminating information to increase awareness and garnering local support. These programs assist in linking caregivers and children to various resources to meet family needs and allow for optimal development, such as housing and nutritious resources, as well as medical or legal assistance (Sege & Harper Browne, 2017; Verbitsky-Savitz et al., 2016). These studies emphasized the importance of such programs as protective factors to minimize or prevent the effects of ACEs.

Coordinated programs assume a more comprehensive approach to address the effects of ACEs and prevent or minimize behaviors that could lead to future ACEs. These programs seek to rectify circumstances that promote the perpetuation of ACEs by creating positive situations that reduce the recurrence and persistent effects of maltreatment. Home based visit programs and parenting classes educate caregivers about ways to develop positive intrafamily relationships, encourage proper development, social connectedness and support, as well as childhood emotional and social competence (Verbitsky-Savitz et al., 2016). Children and adolescents can also benefit from a combined medical and behavioral approach to address mental health and behavioral issues (Asarnow et al., 2015).

Although several community-based programs are being used, few are implemented within health care settings and fewer focus on primary prevention (Dubowitz et al., 2012). Most children attend some type of primary care visits which can provide an opportunity to prevent ACEs. The use of the patient-centered medical home has been promoted to address health disorders and ACEs (Ghandour et al., 2011). Furthermore, evidence suggests that the use of the patient-centered medical home can decrease unnecessary utilization of medical services and hence reduce costs (Nielsen et al,, 2016). The Healthy Steps model is a team-based model that has been shown to be effective for reducing the risk of trauma (Zuckerman et al., 2004).

This study added to previous research by assessing the economic burden of adverse childhood experiences in two states, TN and VA. Further research should extend these results to other states or obtain nationwide estimates for comparison. Although exposure to ACEs and total burden were comparable among the two states, health spending and health costs were quite high in VA compared to TN. This is comparable to a different result which showed the costs of health care in various states in 2021 where the spending per capita TN was $5,982 while the spending per capita in VA was $9,462 (Kaiser Family Foundation, n.d.). An analysis showed that workers in VA paid the largest average family plan premium in the country compared to other states and the third largest average single plan premium; lower-income individuals with employer-sponsored insurance faced a greater burden (Commonwealth Fund, 2019).

### Strengths & Limitations

A key strength of this study was using data from a national survey which allowed for consistency of questions, sampling design and a large enough sample size to increase validity. Analyzing data from two states, enabled the comparison of study findings. The snapshot nature of this cross-sectional study, while unique and convenient, precludes assessment of causality. There is the possibility of recall and social desirability bias as well as the misinterpretation of survey questions due to self-reported data, however the ACE scale has been shown to have good retrospective reporting (Dube et al., 2004). To ensure more generalizable results outside TN and VA, populations and samples from varied geography should be considered and future endeavors should identify possible confounding variables. Due to negative PARs for some conditions, the health burden and spending associated with ACEs were not calculated so we may be underestimating the true impact of exposure to ACEs for all outcomes examined. The negative PARs represent a reduction in the risk factor exposure or outcome resulting in a lesser fraction of the attributable burden. Although the PARs were intended to quantify the relationship between the loss of health within the population and the risks, they only offer a proxy measure to calculate the impact of risk factors or outcomes on ACEs. Also, a comprehensive list of all possible health outcomes was not examined, however we examined the major health outcomes. Risk factors of smoking 100 cigarettes in a lifetime and drinking alcohol within the past 30 days were used in the analysis based on the fact that these benchmarks were used in other literature. However, these benchmarks are quite restrictive and other levels of risk factors should be explored in future analyses.

There is a wide range of DALY values depending on the methodology, however as stated in the methods section, our chosen method follows widely cited recommendations. The use of a uniform value of $235,855 per DALY may ignore the suggestion that the value per DALY is situational. The DALY estimates obtained from the GBD study have a few limitations such as the use of disability weights that may not be suited for high-income countries and the use of models instead of data sets from the states to disintegrate non-fatal estimates by state. Due to the uncertainty regarding the valuation of DALYs, the values are presented separately from health expenses and non-monetized DALYs estimates are also presented. Nonetheless, the findings from this study provide additional knowledge for continued work as this is the first study, to our knowledge, to examine health burden and spending associated with exposure to ACEs in TN and VA.

The ACE tool has been critiqued as not adequately representing the social distribution of adversities and the need for a broader examination of ACEs such as community violence, socioeconomic status and violence. Researchers have described these additional experiences to be important factors that contribute to negative health outcomes (Cronholm et al., 2015; Finkelhor et al., 2015). Thus, future research should utilize surveys that capture some of these measures to fully comprehend how social context affects health and provided updated cost estimates.

## Conclusions

There are various tools to assess exposure to ACEs and methods to mitigate the effects; further research is needed to determine the extent to which the physiological, psychological, and monetary costs can be attenuated through interventions. There is the need for prevention strategies to reduce these effects since resources are scarce and the burden of ACEs described here is enormous. Collaboration between behavioral health, medical health and public health is needed to emphasize integrated care which is essential for the prevention of disorders attributed to ACEs. Health care professionals should integrate screening practices and refer patients to mental health services in order to monitor the development of ACE-attributable disorders. Various ACE screening tools have been created to assess the presence of specific traumas, determine gradient relationships between traumas and outcomes, or use in specific settings. Behavioral health interventions should target early childhood to decrease the effect of ACEs on the life course before children transition into adulthood. Also, providers should address behavioral risk factors related to ACEs.

Trauma informed care practices have been shown to improve health outcomes and enable providers to efficiently care for patients as well as reduce excess costs and avoidable hospital utilization (Center for Health Care Strategies, 2017). Trauma informed care is the framework that recognizes, understands and responds to the symptoms of trauma experienced by individuals that experienced ACEs. This involves understanding the impact of adversity on health and behavior, training staff on best practices, integrating knowledge regarding trauma into procedures and treatment planning, and avoiding re-traumatization by providing patient-care with non-judgmental support (SAMHSA, 2014). A model of trauma-informed systems of care utilized in Northeast Tennessee was created in 2018 and this toolkit is being used nationally for various communities (Haas & Clements, 2018).

Given the high costs of ACEs, it is imperative that stakeholders within the community and policy makers support trauma informed systems of care. There is a national call for policymakers to use results from ACEs studies in order to reinforce intervention efforts and to change delivery systems to bolster comprehensive trauma informed care which supports early intersectoral prevention efforts (Larkin et al., 2014). Screening for ACEs, utilizing trauma informed care as well as leveraging community and clinical interventions can decrease the intergenerational effect of ACEs and avert their burden and costs.

## Data Availability

Available upon request.

## Code Availability

Available upon request.
